# Individualizing Management of Vesicoureteral Reflux

**DOI:** 10.5812/numonthly.1866

**Published:** 2012-06-20

**Authors:** Christopher S. Cooper

**Affiliations:** 1 Department of Urology, University of Iowa Department of Urology, Iowa, United States

**Keywords:** Vesico-Ureteral Reflux, Child, Disease Management

## Abstract

**Background:**

Approaches to the management of vesicoureteral reflux (VUR) in children have changed rapidly in recent years. Multiple studies published over the last decade have contributed to these changes by challenging the dogma that all children with reflux require and benefit from continuous antibiotic prophylaxis. The advent and wide acceptance of endoscopic treatment for VUR has also contributed to these changes. Although new guidelines for VUR management have recently been proposed, they are broad and relatively non-specific. Many physicians and parents remain unsure which children are at risk from their VUR, and which would benefit from antibiotic prophylaxis or surgical intervention.

**Materials and Methods:**

A literature search, followed by an additional search based on bibliographies, was performed for articles reporting on VUR and the utility of antibiotic prophylaxis for its treatment, as well as the chance of spontaneous resolution.

**Results:**

Articles selected for review included those that provided information to assist physicians in determining if a child with VUR is at increased risk of pyelonephritis or persistent VUR, and would benefit from intervention. Particular emphasis was placed on recent prospective, randomized trials in children with VUR.

**Conclusions:**

Because of the multiple factors affecting risk in a child with VUR, specific guidelines for intervention cannot be provided. However, an accurate understanding of these risk factors will help the physician and parents to develop a more individualized management plan for a child with VUR.

## 1. Introduction

The management of vesicoureteral reflux (VUR) has become increasingly controversial over the last decade, as physicians struggle to determine which patients will benefit from the diagnosis and management of this condition (1, 2). Recent guidelines published by the American Urological Association are relatively nonprescriptive, and permit a wide range of management options for most children with VUR (3). These options include observation, continuous antibiotic prophylaxis, endoscopic injection, or open operative correction. Although there are many unanswered questions regarding VUR, there is much data available on VUR. This review is intended to help guide clinicians in the individual management of a child with VUR.

## 2. Antibiotics

Many recent studies have cast doubt on the utility of continuous antibiotics for children with reflux. The use of continuous antibiotics was previously thought to pre-vent bladder infections, and subsequent pyelonephritis and renal scars, in children with VUR. Several large prospective studies had demonstrated that antibiotics were as effective as operative intervention in preventing renal scars, although none of these studies included a control group managed without antibiotics (4, 5). However, in 1997, Reddy randomly assigned a small group of children (n = 29) to treatments of daily antibiotics, no antibiotics, or antibiotics 3 times per week, and found no significant difference in the risk of urinary tract infection (UTI) or renal injury (6). Subsequently, a retrospective study by Cooper et al. in 2000 demonstrated that 51 older children with VUR and normal voiding habits, as well as a minor history of UTIs, could be safely taken off antibiotics despite persistent VUR (7). About 10% of these children developed recurrent UTIs, on average 2.3 years after the antibiotics were discontinued. Subsequent studies, both retrospective and prospective, confirmed that bowel and bladder dysfunction is a major risk factor for developing UTIs either on or off prophylaxis.

In 2001, Thompson demonstrated that a group of 196 children with VUR had the same rate of UTIs and new scar formation when on or off antibiotics (8). Thus, this study also suggested that not all children with VUR benefit from daily antibiotics. However, in 2002, Hellerstein et al. demonstrated that children with grade 3 or greater VUR, as well as voiding dysfunction, had an increased risk of VUR when taken off antibiotics (9). Other retrospective studies also demonstrated that children with grade 3 or greater VUR, as well as bowel or bladder dysfunction, had an increased risk of febrile UTIs (10, 11). The rate of febrile UTIs was about 10%, and they occurred on average 17 months after antibiotics were stopped.

A series of more recent prospective studies have reinforced the conclusion that grade 3 or greater VUR is a risk factor for pyelonephritis and renal scaring in the absence of antibiotics, whereas antibiotics do not seem beneficial in cases of grade 2 or lower VUR. In 2006, Garin et al. demonstrated that 113 children with grades 1–3 VUR, who were randomized to receive or not receive antibiotics, showed no significant differences in susceptibility to UTIs or renal scars (12). In fact, the highest percentage of patients with pyelonephritis in this study comprised those who had VUR and were on antibiotics. In 2008, Pennesi reported on 100 children under 30 months of age with grades 2–4 VUR, diagnosed after pyelonephritis, who were randomized to antibiotics or observation for 2 years, and then all observed without antibiotics for an additional 2 years (13). There was no significant difference in the incidence of pyelonephritis on or off antibiotics (36% vs. 30%, respectively). Dimercaptosuccinic acid (DMSA) scans were noted to be worse in 10 patients, all of whom had grade 4 VUR, which suggests that higher grades of VUR do increase the risk of renal damage. Another prospective randomized trial of antibiotics versus observation in children with grades 1–3 VUR was reported by Roussey-Kesler in 2008 (14). This study showed no overall difference in rates of recurrent UTI or febrile UTI with or without antibiotics. Of note, patients with grade 3 VUR did have a higher risk of recurrent UTI than those with grade 2 VUR (P < 0.01). A similar prospective study that year also demonstrated grade 3 VUR, as well as younger age, to be risk factors for recurrence (15). Recently, the Swedish Reflux Study reported, in a series of papers, the 2-year outcomes of 1-year old children with grades 3 and 4 VUR who were randomized to antibiotic prophylaxis, observation, or endoscopic treatment with Deflux® (Oceana Therapeutics, Edison, NJ) (16). Children were matched for gender, grade of VUR, history of UTIs, and renal defects as demonstrated by DMSA scan. Recurrent UTIs in this group of young children with highgrade reflux occurred most frequently amongst those under surveillance without treatment (17). Fifty-seven percent of the surveillance group had a UTI, which occurred on average 96 days after the study began, whereas only 19% of those on antibiotics had a febrile UTI, and this occurred after 589 days on average. As anticipated, those receiving Deflux® injections had a higher resolution rate; however, the rate of recurrence of reflux within 1 year was 20%, which is similar to other reported rates of VUR recurrence following Deflux® (18). Risk factors for febrile UTI included female gender and persistent VUR. Interestingly, renal damage at entry was not predictive of subsequent UTI or further renal damage (19). Aside from those in the group under surveillance without antibiotics, other patients with increased risk of renal deterioration included those with bowel or bladder dysfunction, and, as expected, those who had febrile UTIs (20). New renal damage was rare in boys.

It is useful to summarize what we have learned from the retrospective and prospective studies reviewed above. Antibiotic prophylaxis appears to provide little benefit for those with grade 2 or lower VUR. Conversely, antibiotic prophylaxis does appear to be beneficial for those with grade 3 or higher VUR, at least among girls. It can also be anticipated that about 15% of children with VUR will have a recurrent febrile UTI within 2 years, and about 15% of these children will develop a renal scar (21). Bowel and/or bladder dysfunction (BBD) is a major risk factor for recurrent UTIs on or off antibiotics, which will occur in about 45% of children with BBD, as opposed to 15% of those without BBD (3). Finally, it is important to remember that a higher grade of VUR is associated with an increased risk of both pyelonephritis and new renal damage (22). The effect of age on the risk of renal damage is not well defined, although many believe that younger children are more susceptible to renal damage from pyelonephritis.

Aside from the questionable efficacy of antibiotic prophylaxis in children with low-grade reflux, there is growing concern about side effects. The most common concern with antibiotic use is the development of resistance.

Multiple studies confirm that exposure to antibiotics increases the likelihood that any subsequent UTIs will be caused by bacteria resistant to the previously prescribed antibiotics (11, 17, 23-25). In general, the risk of resistance appears to be about 3 times greater following treatment with antibiotics.

## 3. Predictors of Resolution

Since both the grade and persistence of VUR affect the risk of pyelonephritis and renal scarring, and it is well known that higher grades of VUR and bilateral VUR are less likely to resolve themselves, information obtained from a cystogram is critical in assessing an individual child’s risk and need for treatment. This information is readily obtained and routinely reported by radiologists performing a voiding cystourethrogram (VCUG). A significant amount of research by the author, however, demonstrates that a cystogram can provide additional predictive information, independent of the patient’s age. One of the most important additional pieces of information, which is readily obtained but rarely reported, is the volume instilled into the bladder at the onset of VUR. This information should be requested by those caring for children with VUR, and should be a routine component of all cystogram reports. This volume, when normalized as a percentage of the child’s age-predicted bladder capacity (PBC = [age in years + 2] × 30 mL), provides a significant prognostic factor for spontaneous resolution of reflux, independent of the grade of VUR (26). Table 1 demonstrates the significant impact that bladder volume at onset of VUR has on resolution rates within 1 or 2 years for a child with grade 2 VUR. Table 2 demonstrates a similar significant impact on resolution rates for a child with grade 3 VUR.

**Table 1:** Prediction of Resolution Rates Based on Predicted Bladder Capacity (PBC) Within 1 or 2 Years for a Child With Grade 2 Vesicoureteral Reflux.

	Volume > 50% PBC at Onset, %		Volume < 50% PBC at Onset, %	
	1, y	2, y	1, y	2, y
Age < 2 y at diagnosis	65	77	37	39
Age > 2 y at diagnosis	60	75	7	7

**Table 2:** Prediction of Resolution Rates Based on Predicted Bladder Capacity (PBC) Within 1 or 2 Years for a Child With Grade 3 Vesicoureteral Reflux.

	Volume > 50% PBC at Onset, %		Volume < 50% PBC at Onset, %	
	1, y	2, y	1, y	2, y
Age < 2 y at diagnosis	80	80	7	14
Age > 2 y at diagnosis	25	63	0	0

**Figure 1:**
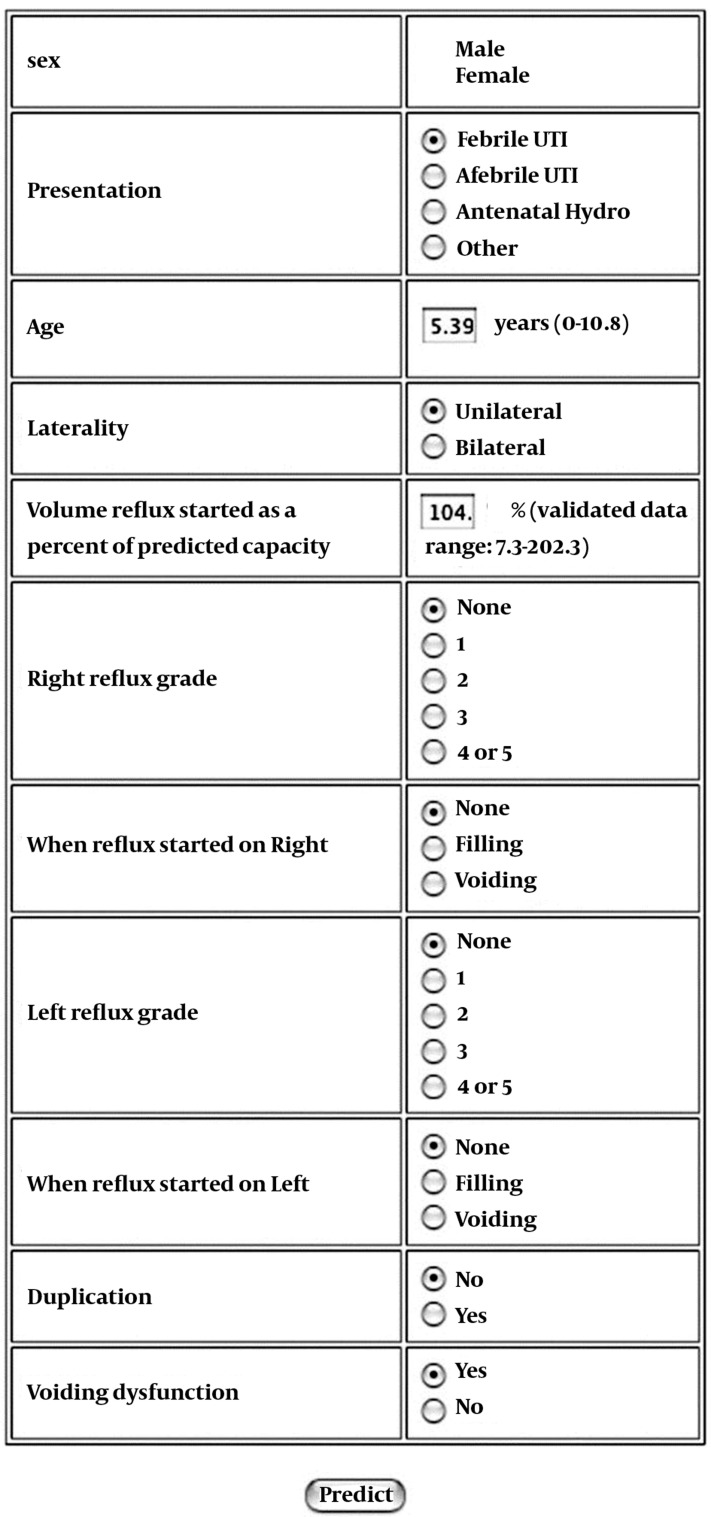
Image of Online Neural Network for Predicting the Chance of 1- and 2-Year Resolution in Children With Vesicoureteral Reflux.

In addition to bladder volume at onset of VUR, grade, laterality, and age have been shown to affect resolution rates, as have several other factors. These include presentation by screening or antenatal hydronephrosis as opposed to presenting after a UTI, the presence of voiding dysfunction, and reflux into a duplicated system (27, 28). Recently it has been shown, as might be expected, that children with improvement on follow-up cystograms are also more likely to resolve their reflux than those without improvement (29). To facilitate the analysis of these multiple independent predictive factors on an individual basis by the busy clinician, the author and colleagues have incorporated them into a computational model, which is readily available online (www.urocomp.net; Figure 1) (30). This model was trained and tested using 205 patients whose age, grade, laterality, gender, presenting symptoms, volume at onset of VUR, presence of voiding dysfunction, ureteral duplication status, and time of occurrence of VUR (during filling or voiding) were known. The accuracy of the model was greater than 80% for both 1- and 2-year predictions of resolution. This model and its accuracy were subsequently validated in a collaborative study of children in Japan (31).

An additional study demonstrated that an abnormal renal scan, as defined by either scars or a relative renal function < 40%, was also a negative prognostic factor for spontaneous reflux resolution independent of grade (32). This has also been shown in infants with grades 3–5 VUR (33). Through the addition of renal scan data, another computer model was generated with an accuracy of 94.5% for prediction of resolution within 2 years (www.urocomp.net) (34). In addition to information obtained from a cystogram, prognostic information may be obtained from renal ultrasound, which is performed on most children diagnosed with VUR. The author recently demonstrated that children with an abnormal renal ultrasound, as defined by the presence of hydronephrosis or a size discrepancy of > 1 cm, had significantly lower 2-year resolution rates than those with a normal renal ultrasound (35). As one might expect, children with an abnormal renal ultrasound were likely to have an abnormal renal scan.

## 4. Conclusions

Almost paradoxically, as more studies provide us with additional information regarding VUR, determining the ideal management of a patient with VUR has become increasingly complex. It is apparent that risk factors for developing recurrent UTI and renal scars must be considered when evaluating the potential benefits of various treatment options. A child with a negligible risk of developing a recurrent febrile UTI is unlikely to benefit from daily antibiotics. In assessing risk, it is important to treat each patient as an individual needing personalized treatment. Since multiple factors affect an individual’s risk, it is not possible to provide excellent healthcare by developing broad, sweeping guidelines that dictate management protocols based on one specific factor, such as grade of reflux. Rather, one must consider additional information, aside from age and grade of reflux, to ultimately provide tailored management. The patient’s history, presenting symptoms, bowel or bladder dysfunction, grade of VUR, likelihood of persistent VUR, and kidney status should all be considered and factored into the determination of a child’s individual risk of developing recurrent febrile UTIs and renal scars. In addition to considering these factors, the physician must also consider the social situation of the child, which, although difficult to quantify, may be one of the greatest predictive factors for a child’s risk of adverse outcome. After assessing these factors, physicians and parents should feel more confident in selecting a treatment plan.
